# *Enterococcus faecalis* EF-2001 protects DNBS-induced inflammatory bowel disease in mice model

**DOI:** 10.1371/journal.pone.0210854

**Published:** 2019-02-28

**Authors:** Eun-Ju Choi, Hyuek Jong Lee, Wan-Jae Kim, Kwon-Il Han, Masahiro Iwasa, Kazumasa Kobayashi, Trishna Debnath, Yujiao Tang, Yi-Sub Kwak, Jin-Hwan Yoon, Eun-Kyung Kim

**Affiliations:** 1 Department of Physical Education, College of Education, Daegu Catholic University, Gyeongsan, Republic of Korea; 2 Center for Vascular Research, Institute for Basic Science (IBS), Daejeon, Republic of Korea; 3 Research & Development Center, Korea BeRM Co. Ltd., Wonju, Republic of Korea; 4 Bien Co. Ltd., Sapporo, Japan; 5 Department of Food Science and Biotechnology, Dongguk University, Goyang, Republic of Korea; 6 Division of Food Bioscience, College of Biomedical and Health Sciences, Konkuk University, Chungju, Republic of Korea; 7 School of Bio-science and Food Engineering, Changchun University of Science and Technology, Changchun, China; 8 Department of Physical Education, Dong-Eui University, Busanjin-gu, Busan, Republic of Korea; 9 Institute for Natural Science Research, Hannam University, Daejeon, Republic of Korea; Toho University Graduate School of Medicine, JAPAN

## Abstract

Recent studies have demonstrated the immunomodulatory effects of heat-killed lactic acid bacteria. The aim of this study was to evaluate the protective effect of heat-killed *Enterococcus faecalis* EF-2001 (EF-2001) on a model of inflammatory bowel disease (IBD). A total of 28 female NC/Nga mice were divided into 4 treatment groups. Controls were fed a normal commercial diet. In the experimental groups, colitis was induced by rectal administration of dinitrobenzene sulfonic acid. Two groups were orally administered 2 and 17 mg/kg EF-2001, respectively. EF-2001 treatment decreased the expression of several cytokines, including cyclooxygenase (COX)-2, inducible nitric oxide synthase (iNOS), interferon (IFN)-γ, interleukin (IL)-1β, and IL-6 in inflamed colon compared to the DNBS alone group. In addition, EF-2001 suppressed DNBS-induced colonic tissue destruction. Therefore, this study strongly suggests that EF-2001 could alleviate the inflammation associated with mouse IBD.

## Introduction

Several probiotic bacteria have been reported as favorable candidates for the treatment and prevention of disease through the regulation of the host immune system [1,2]. Consuming probiotic foods, such as yogurt, has been reported to improve abnormal immune function [3,4]. In addition to live lactic acid bacteria, heat-killed cells also display immunomodulatory functions [5,6]. Inflammatory bowel disease (IBD) is a group of inflammatory disorders of the digestive tract that includes Crohn’s disease (CD) and ulcerative colitis (UC). UC occurs in the innermost lining of the colon and rectum, whereas CD occurs throughout the digestive tract, with inflammation that frequently spreads deep into affected tissues. Clinically, the key symptoms of IBD involve severe diarrhea, pain, fatigue, weight loss and enlargement of lymph nodes [7]. In addition, IBD increases the risk of colon cancer. There is a wealth of evidence that the immune system plays an important role in the development and progression of CD and UC [8,9]. A useful approach to study the pathogenesis and complexity of human IBD is to induce IBD in animals. Widely used models include chemical-induced colitis models such as dinitrobenzene sulfonic acid (DNBS)-induced colitis [10]. DNBS provokes cell-mediated immune responses and prompts transmural inflammation in the gut with morphological and histopathological features similar to human IBD [11].

*Enterococcus faecalis*, a gram-positive commensal bacterium in the guts of mammals, has been reported to have immune regulatory properties [12,13], and strains have been used as probiotics for a variety of beneficial purposes [14]. Heat-killed *E*. *faecalis* derived from the gut have been reported to display a radiation-protective effect, anti-tumor activity, and anti-atopic dermatitis properties [15–17]. Heat-killed *E*. *faecalis* can therefore be used safely without risk of infection or antibiotic resistance. However, little is known about the effects of heat-killed *E*. *faecalis* on an IBD model. Therefore, the goal of this study was to examine the effect of heat-killed probiotic *E*. *faecalis* EF-2001 (EF-2001) on a mouse model of DNBS-induced colitis.

## Materials and methods

### Preparation of EF-2001

EF-2001 is a commercially available probiotic that was originally isolated from healthy human infant feces. It was supplied as a heat-killed, dried powder by Nihon BRM Co. Ltd (Tokyo, Japan). One gram of dried EF-2001 is equivalent to over 7.5 × 10^12^ colony-forming units prior to being heat-killed.

### Experimental design and induction of colitis

**Fig 1:**
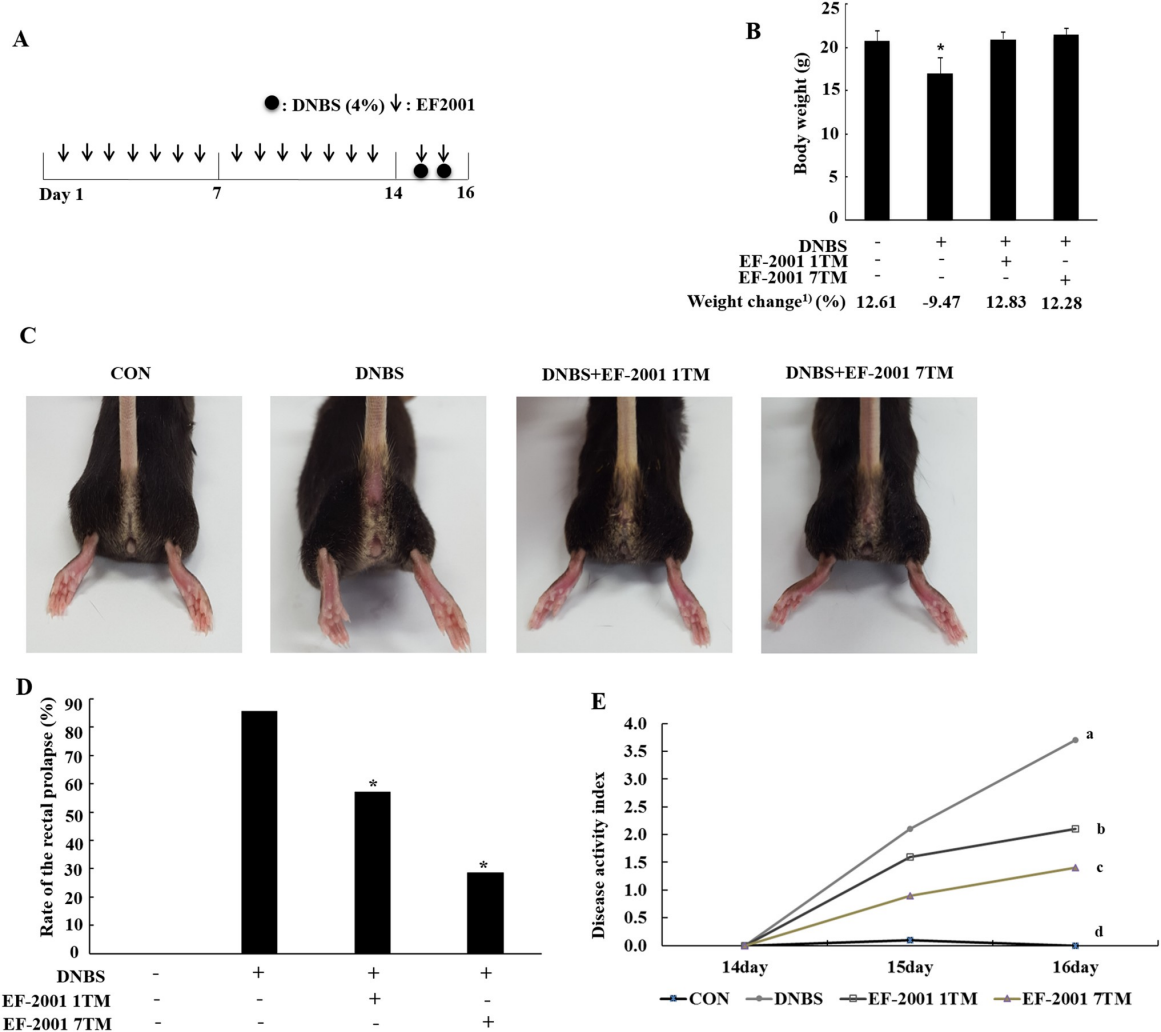
Experimental design (A). The weight of individual mice was measured daily (B). Representative images of mouse anuses from each group (C). CON (fed commercial diet), (D). Rate of the rectal prolapse (%), (E). Disease activity index, DNBS (induced colitis by dinitrobenzene sulfonic acid, DNBS), EF-2001 1TM (EF-2001 1TM administration + induced colitis by DNBS), EF-2001 7TM (EF-2001 7TM administration + induced colitis by DNBS). *, *p*<0.05 compared to the control group. ^1)^Weight change as percent of weight gain(+) or loss(-) from the start of the experiment. ^a-d^Means are significantly different at *p* < *0*.*05*.

Four-week-old female NC/Nga mice (average body weight, 18.7 ± 1.2 g) were obtained from Orient Bio (Seongnam, Korea). Animals were kept in individual stainless steel bottomed cages in a windowless room on a 12 h light/dark cycle. Commercial diet and sterilized water were provided ad libitum throughout the experiment. After 7 days of adaptation, the 28 mice were divided in to 4 groups (n = 7 mice/group): control mice fed a normal commercial diet; mice with DNBS-induced by rectal administration of colitis; mice with DNBS-induced by rectal administration of colitis treated by oral administration of 2 mg/kg (1TM) EF-2001; and mice with DNBS-induced by rectal administration of colitis treated by oral administration of 17 mg/kg/day (7TM) EF-2001). The mice in the control and DNBS groups were fed a commercial diet and water. The EF-2001 treatment groups were administrated orally and daily gavage doses of EF-2001 for 16 days, then colitis was induced in the three experimental groups by rectal administration of 4% DNBS in 50% ethyl alcohol for two days (Fig 1A). The dosages of EF-2001 were determined by previous reports [17,18]. All animal care procedures and experiments were approved by the Institutional Animal Care and Use Committee of Konkuk University (KU15113). Upon completion of the treatment period, mice were euthanized and the colons were removed and placed in ice-cold phosphate-buffered saline (PBS) for histological and expression analysis.

### Disease activity index

During the DNCB treatment, a disease activity index (DAI) score can be assessed to evaluate the clinical progression of colitis. The DAI is the combined score of weight loss compared to initial weight, stool consistency, and bleeding. Scores are defined as follows: weight loss: 0 (no loss), 1 (1–5%), 2 (5–10%), 3 (10–20%), and 4 (>20%); stool consistency: 0 (normal), 2 (loose stool), and 4 (diarrhea); and bleeding: 0 (no blood), 1 (Hemoccult positive), 2 (Hemoccult positive and visual pellet bleeding), and 4 (gross bleeding, blood around anus) [18].

### Histological study

Colons were cut to approximately 1 cm^3^ in size and placed in Eppendorf tubes containing 5 mL 4% paraformaldehyde in PBS for overnight fixation. Afterward, the colon tissues were placed in a histological cassette and transferred through baths of progressively more concentrated ethanol to remove the water. The ethanol was removed and replaced with the hydrophobic clearing agent xylene. This was replaced with molten paraffin wax, which acts as an infiltration agent. After embedding, the tissue were prepared as sections (thickness: 10 μm) and stained with hematoxylin and eosin for histological analysis.

Histological score, crypt architecture (normal, 0—severe crypt distortion with loss of entire crypts, 3), degree of inflammatory cell infiltration (normal, 0 –dense inflammatory infiltrate, 3) [19].

### Real-time polymerase chain reaction (PCR)

Quantitative real-time PCR was carried out using a Thermal Cycler Dice TP850 (Takarabio Inc., Shiga, Japan) according to the manufacturer’s protocol. Total RNA was isolated from colons from each group using TRIzol. First-strand complementary DNA (cDNA) was synthesized using Superscript II reverse transcriptase (Invitrogen, Carlsbad, CA, USA). cDNA synthesis was performed at 45°C for 60 min, followed by RT inactivation at 95°C for 5 min. The conditions for PCR were similar to those previously described [16]. Briefly, 2 μL of cDNA (100 ng), 1 μL each of sense and antisense primer solutions (0.4 μM), 12.5 μL of SYBR Premix Ex Taq (Takara Bio Inc.), and 9.5 μL of dH_2_O were mixed to obtain a final 25-μL reaction mixture. The primers used for qPCR were as follows: mouse iNOS forward primer: ATC ATG AAC CCC AAG AGT TT, reverse primer: AGA GTG AGC TGG TAG GTT CC; mouse COX-2 forward primer: AAG ACT TGC CAG GCT GAA CT, reverse primer: CTT CTG CAG TCC AGG TTC AA; IFN-γ forward primer: TCA AGT GGC ATA GAT GTG GA, reverse primer: TGG CTC TGC AGG ATT TTC AT; mouse IL-1β forward primer: AAC CAA GCA ACG AVA AAA TA, reverse primer: AGG TGC TGA TGT ACC AGT TG; mouse IL-6 forward primer: CCG GAG AGG AGA CTT CAC AG, reverse primer: GGA AAT TGG GGT AGG AAG GA; mouse IL-10 forward primer: TAA GGC TGG CCA CAC TTG AG, reverse primer: GTT TTC AGG GAT GAA GCG GC; and mouse glyceraldehyde 3-phosphate dehydrogenase (GAPDH) forward primer: GCA CAG TCA AGG CCG AGA AT, reverse primer: GCC TTC TCC ATG GTG GTG AA.

The amplification conditions were 10 s at 95°C, then 40 cycles of 5 s at 95°C and 30 s at 60°C, 15 s at 95°C, 30 s at 60°C, and 15 s at 95°C. The mRNA levels of the target genes, relative to GAPDH, were normalized using the following formula: relative mRNA expression = 2^−(Ct of target gene −Ct of GAPDH)^, where Ct is the threshold cycle value. For each sample, the expression of the analyzed gene was normalized to that of GAPDH and presented as the relative mRNA level.

### Statistical analysis

All results were expressed as mean ± standard deviation (SD). Statistical analyses were performed with Graph Pad Prism version 5.00 for windows (GraphPad Software, La, Jolla, CA, USA). The observed differences were analyzed for statistical significance by one-way analysis of variance with Tukey’s multiple comparison as a post-hoc test. Differences were considered significant at p < 0.05.

## Results

### Effect of EF-2001 on body weight, rectal prolapse, and colon and mesenteric lymph node size

**Fig 2:**
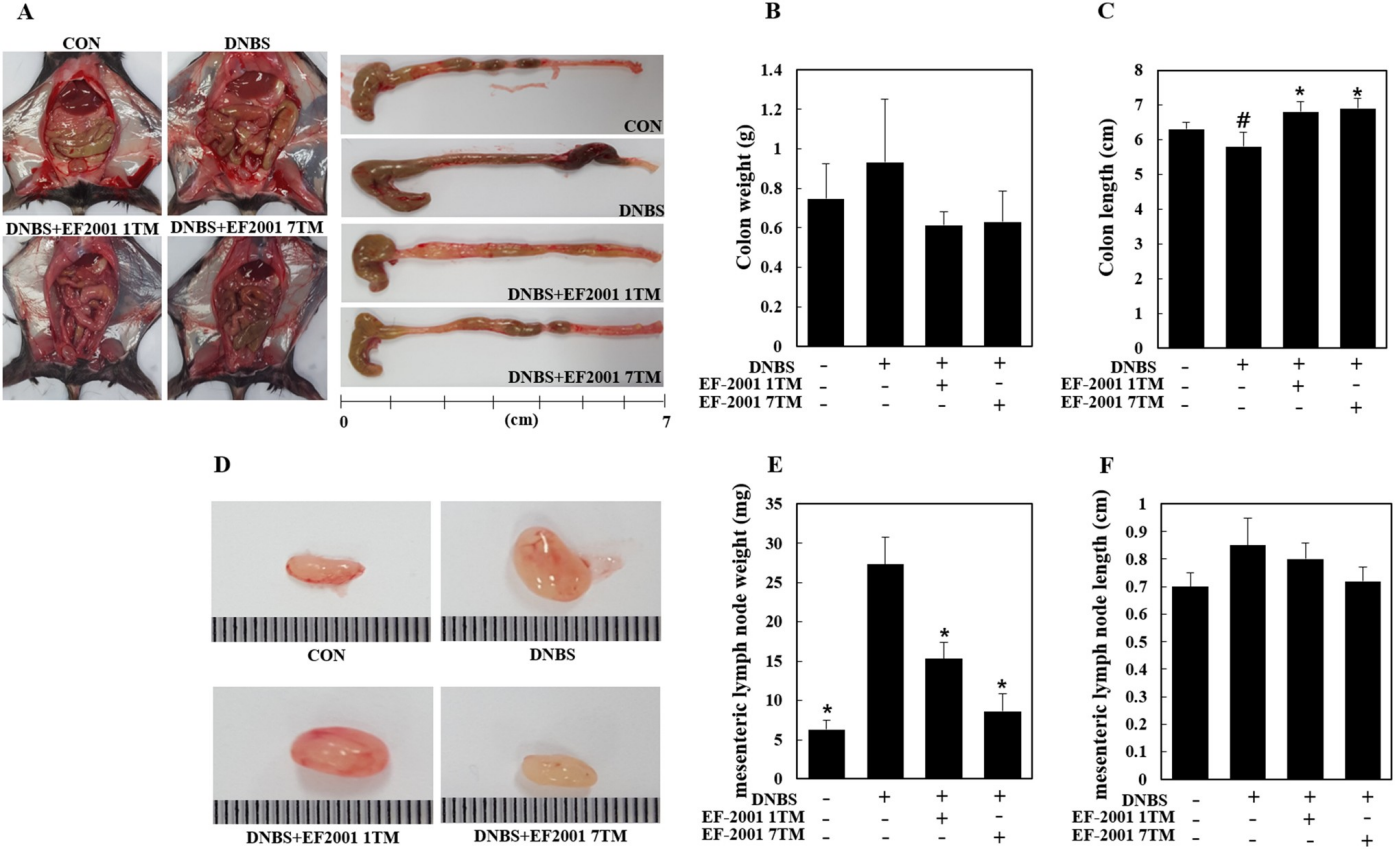
Effect of EF-2001 on the colon and mesenteric lymph node. Representative images of mouse colons from each group (A). Weight and length of mouse colons (B,C). Mesenteric lymph node size, weight, and length (D,E,F). CON (fed commercial diet), DNBS (induced colitis by dinitrobenzene sulfonic acid, DNBS), EF-2001 1TM (EF-2001 1TM administration + induced colitis by DNBS), EF-2001 7TM (EF-2001 7TM administration + induced colitis by DNBS). ^#^, *p*<0.05 compared to the control group. *, *p*<0.05 compared to the DNBS-only group.

We monitored body weight for each group of mice daily during the experimental period. Body weight was reduced after rectal supplementation with DNBS, however the weights of DNBS+EF-2001 1TM and DNBS+EF-2001 7TM groups (Fig 1B) were recovered with EF-2001 administration. Rectal prolapse was also observed in the DNBS+EF-2001 1TM and DNBS+EF-2001 7TM groups, but was improved compared to DNBS-alone mice (Fig 1C and 1D). Furthermore, in the disease activity index, we found the DNBS+EF-2001 1TM and DNBS+EF-2001 groups disease activity scorings were lower than DNCB group (Fig 1E). After the experimental period, mice were euthanized and the length and weight of the colon and mesenteric lymph node were measured. The colon weight did not significantly differ between groups (Fig 2A and 2B). However, the DNBS group colons were shorter than those of the EF-2001 1TM- and EF-2001 7TM-treated groups (Fig 2A and 2C). We observed the increased-mesenteric lymph nodes in the DNBS group compared with the control group, and EF-2001 treatment reduced mesenteric lymph node weight compared to the DNBS group (Fig 2D and 2E).

### Effect of EF-2001 on cytokine expression in the mouse colon

**Fig 3:**
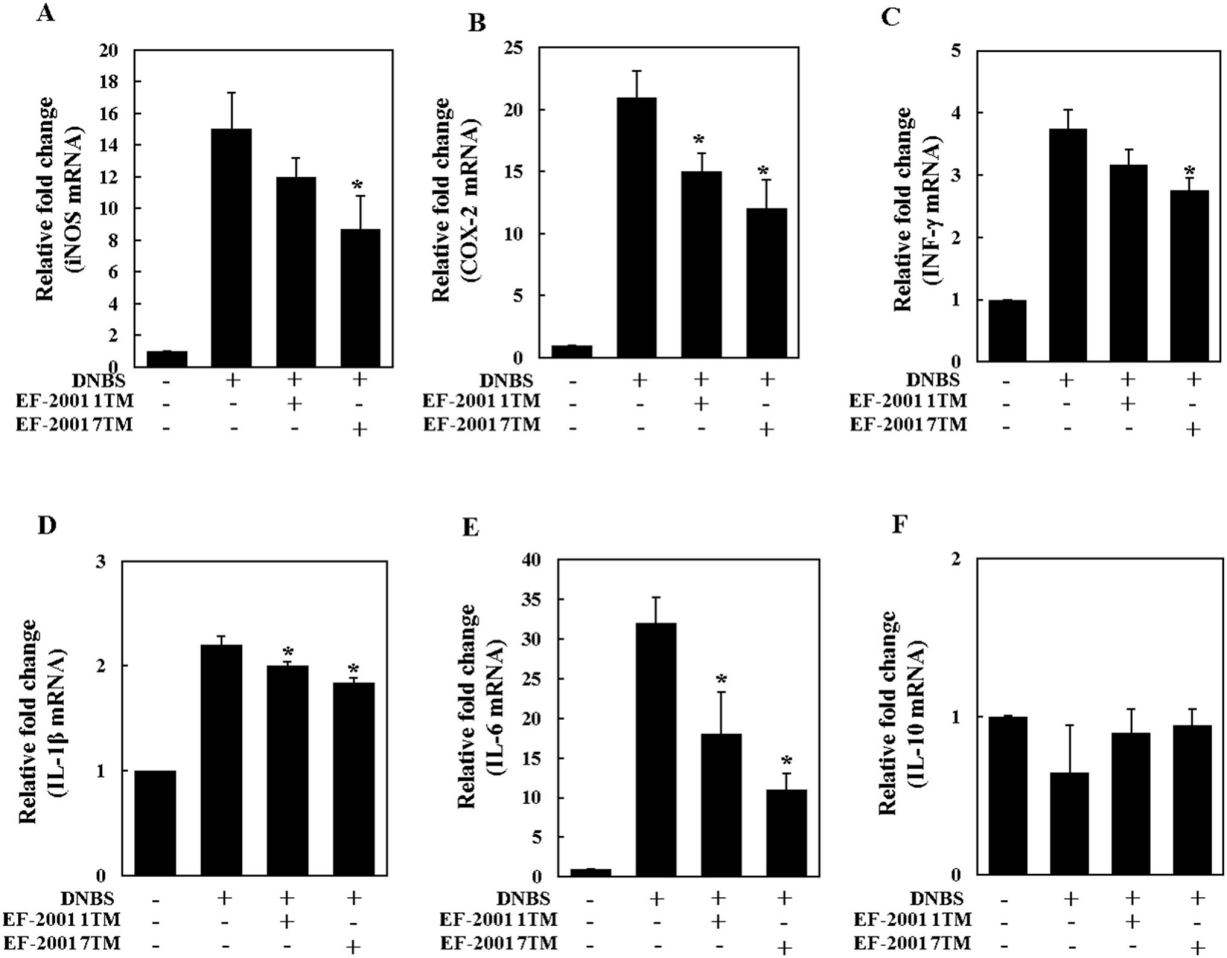
Cytokine expression in the mouse colon. DNBS (induced colitis by dinitrobenzene sulfonic acid, DNBS), EF-2001 1TM (EF-2001 1TM administration + induced colitis by DNBS), EF-2001 7TM (EF-2001 7TM administration + induced colitis by DNBS). *, *p*<0.05 compared to the DNBS-only group.

Colon expression of the cytokines inducible nitric oxide synthase (iNOS) and cyclooxygenase (COX)-2 was much higher in the DNBS group compared to the control group. The expression of these cytokines was decreased in the DNBS+EF-2001 1TM and DNBS+EF-2001 7TM groups, indicating that EF-2001 suppressed the DNBS-induced overexpression of iNOS and COX-2 (Fig 3A and 3B). In addition, expression of IFN-γ, IL-1β and IL-6 was increased in the DNBS group compared to the control group, and significantly inhibited by EF-2001 treatment (Fig 3C–3E). However, there were no significant changes in IL-10 expression between the groups. Meanwhile, the expression of IL-10 in the DNBS group exhibited pretty higher SD values than others, it was due to one outlier value. In addition, from the results, EF-2001 didn’t affect anti-inflammatory cytokine IL-10, which inhibits lipopolysaccharide and bacterial product mediate induction of the pro-inflammatory cytokines IFN-γ and IL-1β [20,21].

### Effect of EF-2001 on mouse colon histology

**Fig 4:**
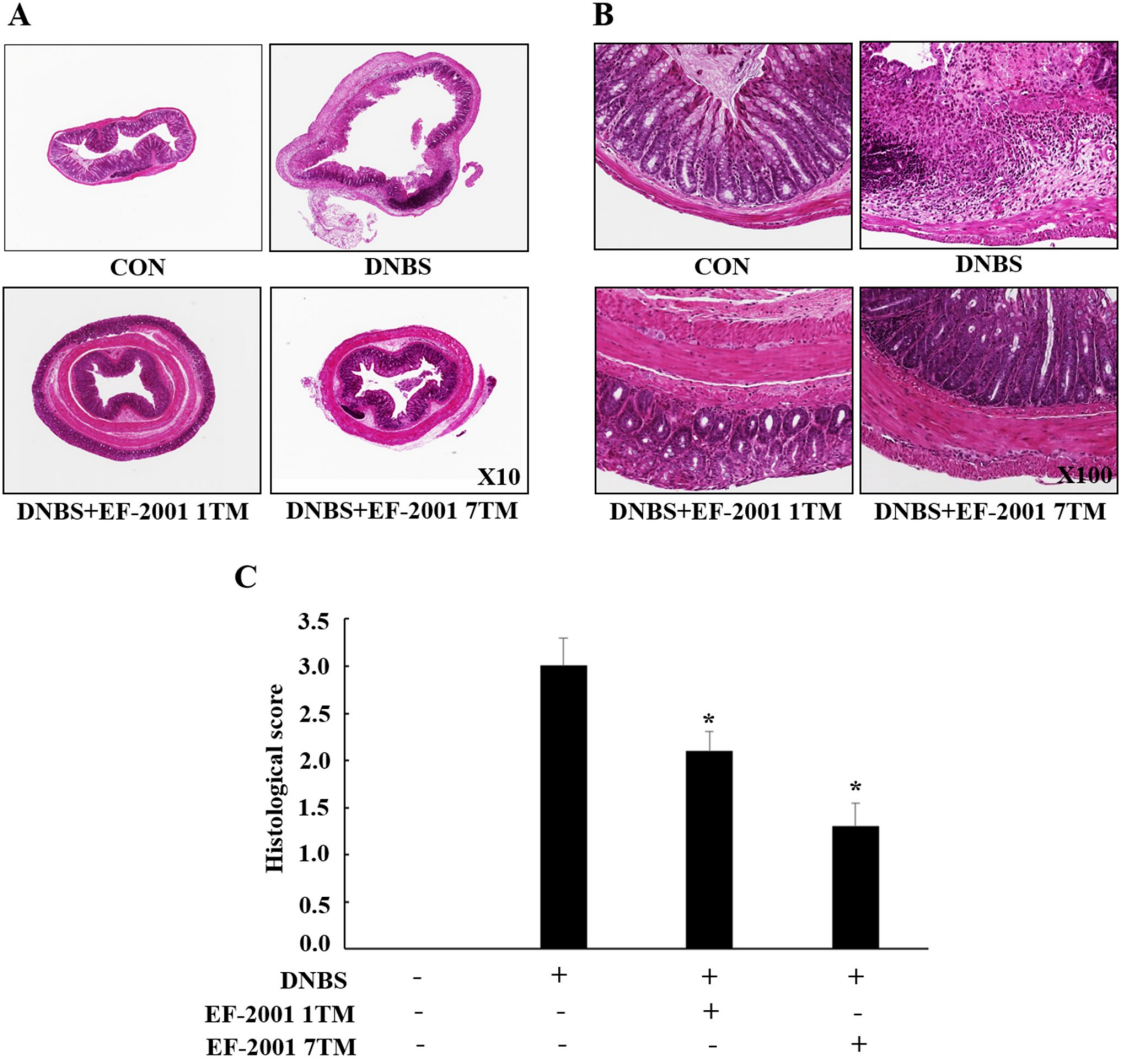
Effect of EF-2001 on the histopathological outcomes of DNBS-induced colitis. Colon sections from euthanized mice were stained with hematoxylin and eosin. Representative tissue sections of DNBS (induced colitis by dinitrobenzene sulfonic acid, DNBS), EF-2001 1TM (EF-2001 1TM administration + induced colitis by DNBS), and EF-2001 7TM (EF-2001 7TM administration + induced colitis by DNBS) mice imaged with 10×(A) and 100× (B) objectives, (C) Histological score.

Control group showed normal appearing colonic crypts that are uniform and evenly placed. However, extensive epithelial damage and crypt destruction were observed in colonic tissue sections from mice in the DNBS group (Fig 4). The DNBS groups showed crypts that are shorter and more variable in length and luminal diameter, and showed focal crypt dropout, goblet cell depletion, evidence of epithelial regeneration and restitution, and an infiltrate of acute inflammatory cells extending into the submucosa. On the contrary, these symptoms were protected by EF-2001 treatment, as observed in tissue sections from the DNBS+EF-2001 1TM and DNBS+EF-2001 7TM groups (Fig 4A and 4B), and the histological score in Fig 4C. DNBS group (3.0±0.3) exhibited the highest score than DNBS+EF-2001 1TM (2.1±0.2) and DNBS+EF-2001 7TM (1.3±0.3) groups.

## Discussion

In recent years, major progress has been made in both the diagnosis and treatment of IBD, resulting in a better quality of life for individuals affected by CD and ulcerative colitis. The probiotics—microorganisms with preventive and/or therapeutic potential may be reduced the duration of active disease [22, 23]. Recently, probiotics such as Streptococcus thermophilus NCIMB 41856, Lactobacillus plantarum CGMCC1258, and Lactobacillus delbrueckii TUA4408L showed beneficial anti-inflammatory effects in inflammatory bowel disease [24,25]. However, when the microorganism makes a change from commensal to pathogenic, and migrate from the gastrointestinal tract to the bloodstream potentially, it can lead to life-threatening infections, such as sepsis or bacterial endocarditis [26,27]. Although the treatment benefits of probiotics have been reported for more than 100 years ago, the safety issues have not yet been clearly identified. There are case reports of complications from certain bacteria treatments that means that the probiotic safety should be reconsidered [27–29]. In individuals with severe immunodeficiency or a pre-existing structural heart disease, lactic acid bacteria can shift and cause serious infection [30–32]. In contrast, non-viable microbial or microorganisms cell extracts have no shelf-life problems and can reduce the risks of microbial translocation and infection associated with live probiotics. In particular, non-viable or heat-sterilized lactobacilli provide biological activities as well as advantages of extended product shelf life, convenient transportation and easier storage [33]. However, the health benefits of non-viable probiotics for IBD are not well known. Therefore, in this context, we have used heat-killed *E*. *faecalis* to treat colonic injury in mice induced by rectal supplementation with DNBS. DNBS-induced colitis is a widely used model that is phenotypically similar to colitis in humans, including loss of body weight, mucosal ulceration, and colonic shortening [34]. We monitored body weight, rectal prolapse, and colon and mesenteric lymph node size. EF-2001 did not affect body or colon weight, or mesenteric lymph node length; however, it affected rectal prolapse, colon length, and mesenteric lymph node weight.

Cytokines are significantly involved in the pathogenesis of IBD, and iNOS and COX-2 are responsible for initiating, regulating, and perpetuating inflammation in IBD. Therefore, we measured the expression of iNOS and COX-2 in inflamed colons by real time PCR, and found that EF-2001 inhibited their expression. Previous studies have reported that colonic epithelial cells are principally responsible for gut NO production, and elevated iNOS activity was found in the colons of patients with UC [35–38]. Furthermore, Singer et al [39] observed iNOS expression in the inflamed colons of patients with IBD, consistent with our results in infectious colitis.

High IFN-γ production, as part of a Th1-driven immune response, has been related to colitis in mice [40]. In addition, Ito et al [41] reported that IFN-γ played a fundamental role in the initiation of colitis in mice. Further, IFN-γ activates downstream effector cells to produce pro-inflammatory cytokines such as IL-1β. Therefore, suppression of IFN-γ and IL-1β induction may explain the anti-inflammatory properties observed for EF-2001. In addition, the Th1 or humoral response is critical for resistance against extracellular pathogens, and these cells produce certain IL-family cytokines, including IL-1β, IL-6, and IL-10. In patients with UC, the pattern of cytokine expression varies from that seen in CD, with an increased level of IL-6 [42]. We observed increased IL-6 expression in the DNBS group. However, the DNBS+EF-2001 1TM and DNBS+EF-2001 7TM groups displayed decreased IL-6 expression, suggesting that production of other Th2-related cytokines may also be affected. IL-10 is also a well-known anti-inflammatory cytokine, with inhibitory effects on autoimmune disease [43]. However, unlike IL-6, production of IL-10 was down-regulated in DNBS group (no significance), and no significant differences in IL-10 expression were observed between the groups in our study. This result was similar with Wu et al [44]. They reported that IL-10 was significantly inhibited in trinitrobenzene sulfonic acid (TNBS)-induced IBD model of mice.

Histological analysis revealed extensive damage to the epithelium along with crypt destruction in colonic tissue sections from the DNBS group. On the contrary, crypt destruction was attenuated in the DNBS+EF-2001 1TM and DNBS+EF-2001 7TM groups. This data is similar to a previous study by Morampudi et al [45]. They demonstrated that a reduced histological damage including crypt abscess formation, inflammatory cell infiltration, and loss of mucosal integrity in fish oil fed rats from DNBS colitis models.

## Conclusions

The present study demonstrates the ability of EF-2001 to protect from DNBS-induced colitis. EF-2001 attenuated IBD symptoms, suppressing the pathogenic shortening of colon length, reducing mesenteric lymph node weight, and downregulating proinflammatory cytokine expression in the colon, thereby improving DNBS-induced colonic tissue destruction. These results clearly demonstrate that therapeutic use of EF-2001 may reduce inflammation associated with colitis. However, in this study there were no groups for heat killed or live EF-2001 without administration of DNBS, and live EF-2001 with administration of DNBS. Therefore further study is strongly suggested to demonstrate the difference of the heat killed and live EF-2001 on IBD.

## Supporting information

S1 File Supporting information file include body weigh for Fig 1B. (XLSX)

## References

[1] Kim NY, Ji GE. Effects of probiotics on the prevention of atopic dermatitis. Korean J Pediatr. 2012; 55: 193–201. 10.3345/kjp.2012.55.6.193 22745643 PMC3382699

[2] Roessler A, Friedrich U, Vogelsang H, Bauer A, Kaatz M, Hipler UC, et al The immune system in healthy adults and patients with atopic dermatitis seems to be affected differently by a probiotic intervention. Clin Exp Allergy. 2001; 107: 129–134. 10.1111/j.1365-2222.2007.02876.x 18028460

[3] Schiffrin EJ, Parlesak A, Bode C, Bode J, vant Hof AM, Grathwohl D, et al Probiotic yogurt in the elderly with intestinal bacterial overgrowth: endotoxaemia and innate immune functions. Br J Nutr. 2009; 101: 961–966. 19353762 10.1017/s0007114508055591

[4] Chaves S, Perdigon G, De Moreno De Leblanc A. Yoghurt consumption regulates the immune cells implicated in acute intestinal inflammation and prevents the recurrence of the inflammatory process in a mouse model. J Food Prot. 2011; 74: 801–811. 10.4315/0362-028X.JFP-10-375 21549052

[5] Liu CF, Tseng KC, Chiang SS, Lee BH, Hsu WH, Pan TM. Immunomodulatory and antioxidant potential of Lactobacillus exopolysaccharides. J Sci Food Agric. 2011, 91: 2284–2291. 10.1002/jsfa.4456 21560134

[6] Kawase M, He F, Kubota A, Yoda K, Miyazawa K, Hiramatsu M. Heat-killed Lactobacillus gasseri TMC0356 protects mice against influenza virus infection by stimulating gut and respiratory immune responces. FEMS Immunol Med Microbiol. 2012, 64: 280–288. 10.1111/j.1574-695X.2011.00903.x 22098223

[7] Horsthuis K, Stokkers PC, Stoker J. Detection of inflammatory bowel disease: diagnostic performance of cross-sectional imaging modalities. Abdom. Imaging. 2008, 33: 407–416. 10.1007/s00261-007-9276-3 17619923 PMC2386533

[8] Kaser A, Zeissig S, Blumberg RS. Inflammatory Bowel Disease. Annu Rev Immunol. 2010, 28: 573–621. 10.1146/annurev-immunol-030409-101225 20192811 PMC4620040

[9] Baumgart DC, Sandborn WJ. Inflammatory bowel disease: clinical aspects and established and evolving therapies. Lancet. 2007, 369: 1641–1657. 10.1016/S0140-6736(07)60751-X 17499606

[10] Morampudi V, Bhinder G, Wu X, Dai C, Sham HP, Vallance BA, et al DNBS/TNBS colitis models: providing insights into inflammatory bowel disease and effects of dietary fat. J Vis Exp. 2014, 84: e51297. 10.3791/51297 PMC4140598 24637969

[11] de Almeida AB, Sánchez-Hidalgo M, Martín AR, Luiz-Ferreira A, Trigo JR, Vilegas W, et al Anti-inflammatory intestinal activity of Arctium lappa L. (Asteraceae) in TNBS colitis model. J Ethnopharmacol. 2013, 146: 300–310. 10.1016/j.jep.2012.12.048 23313393

[12] Cai H, Chen J, Liu J, Zeng M, Ming F, Lu Z, et al CRIP1, a novel immune-related protein, activated by Enterococcus faecalis in porcine gastrointestinal epithelial cells. Gene 2017, 598: 84–96. 10.1016/j.gene.2016.11.009 27836662

[13] Inoue R, Tsukahara T, Matsukawa N, Watanabe T, Bukawa W, Nakayama K, et al Rapid induction of an immune response in rat Peyer’s patch after oral administration of Enterococcus faecalis strain EC-12. Biosci Biotechnol Biochem. 2013, 77: 863–866. 10.1271/bbb.120838 23563541

[14] Fritzenwanker M, Kuenne C, Billion A, Hain T, Zimmermann K, Goesmann A, et al Complete genome sequenc of the probiotic Enterococcus faecalis Symbioflor1 Clone DSM 16431. Genome Announc. 2013, 1: e00165–12. 10.1128/genomeA.00165-12 PMC3569346 23405346

[15] Gu YH, Iwasa M, Iwasa H, Kobayashi K, Itokawa Y, Ishida T. Radiation protection effect for EF2001 (Enterococcus faecalis 2001). Med Biol. 2007, 151: 289–295.

[16] Yanagisawa T, Gu YH, Tsuchihashi E, Umekawa M, Yamamoto H, Iwasa T, et al Analgesic and anti-neoplastic effects of the immunization-active fraction of Enterococcus faecalis 2001. J Orient Med. 2000, 5: 97–102.

[17] Choi EJ, Iwasa M, Han KI, Kim WJ, Tang YJ, Hwang YJ, et al heat-killed Enterococcus faecalis EF-2001 ameliorates atopic dermatitis in a murine model. Nutrients. 2016, 8: 146 10.3390/nu8030146 26959058 PMC4808875

[18] Choi EJ, Iwasa M, Han KI, Kim WJ, Tang YJ, Han WC, et al Effect of *Enterococcus faecalis* EF-2001 on experimentally induced atopic eczema in mice. Food Sci. Biotechnol. 2016, 25: 1087–1093. 10.1007/s10068-016-0175-7 30263379 PMC6049111

[19] Kim JJ, Shajib MS, Manocha MM, Khan WI. Investigating intestinal inflammation in DSS-induced model of IBD. Journal of visualized experiments: J.V.E. 2012, 60. 10.3791/3678 PMC3369627 22331082

[20] Cooper HS, Murthy SN, Shah RS, Sedergran DJ. Clinicopathologic study of dextran sulfate sodium experimental murine colitis. Lab. Invest. 1993, 69: 238–249. 8350599

[21] Varma TK, Toliver-Kinsky TE, Lin CY, Koutrouvelis AP, Nichols JE, Sherwood ER. Cellular mechanisms that cause suppressed gamma interferon secretion in endotoxin-tolerant mice. Infect Immun. 2001, 69: 5249–5263. 10.1128/IAI.69.9.5249-5263.2001 11500393 PMC98633

[22] Opp MR, Smith EM, Hughes TK. Interleukin-10 (cytokine synthesis inhibitory factor) acts in the central nervous system of rats to reduce sleep". J Neuroimmunol. 1995, 60: 165–168. 7642744 10.1016/0165-5728(95)00066-b

[23] Rioux M. A. Bacillus subtilis as a Probiotic: Implications for Inflammatory Bowel Disease and Intestinal Colonization of Candida albicans (Doctoral dissertation, Worcester Polytechnic Institute).

[24] Vanderpool C, Yan F, Polk D.B. Mechanisms of probiotic action: Implications for therapeutic applications in inflammatory bowel diseases. Inflamm. Bowel Dis. 2008, 14: 1585–1596. 10.1002/ibd.20525 18623173

[25] Bailey JR, Vince V, Cogan TA, Streptococcus thermophilus NCIMB 41856 ameliorates signs of colitis in an animal model of inflammatory bowel disease. Benef Microbes. 2017, in press 10.3920/BM2016.0110 28618865

[26] Plaza-Díaz J, Ruiz-Ojeda FJ, Vilchez-Padial LM, Gil A. Evidence of the anti-inflammatory effects of probiotics and synbiotics in intestinal chronic diseases. Nutrients. 2017, 9: E555 10.3390/nu9060555 28555037 PMC5490534

[27] Kanasugi H, Hasegawa T, Yamamoto T, Abe S, Yamaguchi H. Optimal dose of Enterococcal preparation(FK-23) supplemented per orally for stimulation of leukocyte reconstitution in dogs treated with cyclophosphamide. J Vet Med Sci. 1996, 56: 563–565. 10.1292/jvms.58.563 8811628

[28] Satonaka K, Ohashi K, Nohmi T, Yamamoto T, Abe S, Uchida K, et al Prophylactic effect on Enterococcus faecalis FK-23 preparation on experimental candidiasis in mice. Microbiol Immunol. 1996, 40: 217–222. 8934676 10.1111/j.1348-0421.1996.tb03337.x

[29] Mason KL, Stepien TA, Blum JE, Holt JF, Labbe NH, Rush JS, et al From commensal to pathogen: Translocation of Enterococcus faecalis from the midgut to hemocoel of Manduca sexta. mBio. 2011, 2: e00065–11. 10.1128/mBio.00065-11 21586646 PMC3101781

[30] Fisher K, Phillips C. The ecology, epidemiology and virulence of Enterococcus. Microbiology. 2008, 155: 1749–1757. 10.1099/mic.0.026385-0 19383684

[31] Metchnikoff E. Essais optimists. The prolongation of life: Optimistic studies In classics in longevity and aging (Series); Mitchell P.C., Metchnikoff I.I., Eds, Springer: Putnam, NY, USA, 1908.

[32] Besselink MG, van Santvoort HC, Buskens E, Boermeester MA, van Goor H, Timmerman HM, et al Probiotic prophylaxis in predicted severe acute pancreatitis: A randomised, double-blind, placebo-controlled trial. Lancet. 2008, 371: 651–659. 10.1016/S0140-6736(08)60207-X 18279948

[33] Kochan P, Chmielarczyk A, Szymaniak L, Brykczynski M, Galant K, Zych A, et al Lactobacillus rhamnosus administration causes sepsis in a cardiosurgical patient—Is the time right to revise probiotic safety guidelines? Clin Microbiol Infect. 2011, 17: 1589–1592. 10.1111/j.1469-0691.2011.03614.x 21848974

[34] Aguirre M, Collins MD. Lactic acid bacteria and human clinical infection. J Appl Bacteriol. 1993, 75: 95–107. 8407678 10.1111/j.1365-2672.1993.tb02753.x

[35] Ou CC, Lin SL, Tsai JJ, Lin MY. Heat-killed lactic acid bacteria enhance immunomodulatory potential by skewing the immune response toward Th1 polarization. J Food Sci. 2011, 76: M260–M267. 10.1111/j.1750-3841.2011.02161.x 22417436

[36] Ursino MG, Vasina V, De Ponti F. Protection from DNBS-induced colitis by the tachykinin NK(1) receptor antagonist SR140333 in rats. Eur. J. Pharmacol. 2009, 603: 133–137. 10.1016/j.ejphar.2008.11.064 19103194

[37] Middleton SJ, Shorthouse M, Hunter JO. Increased nitric oxide synthesis in ulcerative colitis. Lancet. 1993, 341: 465–466. 8094492 10.1016/0140-6736(93)90211-x

[38] Boughton-Smith NK, Evans SM, Hawkey CJ, Cole AT, Balsitis M, Whittle BJ, et al Nitric oxide synthase activity in ulcerative colitis and Crohn’s disease. Lancet. 1993, 342: 338–340. 7687730 10.1016/0140-6736(93)91476-3

[39] Lundberg JO, Hellstrom PM, Lundberg JM, Alving K. Greatly increased luminal nitric oxide in ulcerative colitis. Lancet. 1994, 344: 1673–1674. 7996962 10.1016/s0140-6736(94)90460-x

[40] Singer II, Kawka DW, Scott S, Weidner JR, Mumford RA, Riehl TE, Stenson WF. Expression of inducible nitric oxide synthase and nitrotyrosine in colonic epithelium in inflammatory bowel disease. Gastroenterology. 1996, 111: 871–885. 8831582 10.1016/s0016-5085(96)70055-0

[41] Debnath T, Kim DH, Lim BO. Natural products as a source of anti-inflammatory agents associated with inflammatory bowel disease. Molecules. 2013, 18: 7253–7270. 10.3390/molecules18067253 23783459 PMC6270544

[42] Ito R, Shin-Ya M, Kishida T, Urano A, Takada R, Sakagami J, et al Interferon-gamma is causatively involved in experimental inflammatory bowel disease in mice. Clin Exp Immunol. 2006, 146: 330–338. 10.1111/j.1365-2249.2006.03214.x 17034586 PMC1942055

[43] Madsen K. Combining T cells and IL-10: a new therapy for Crohn’s disease? Gastroenterology. 2002, 123: 2140–2144. 10.1053/gast.2002.37289 12454869

[44] Saraiva M, O’Garra A. The regulation of IL-10 production by immune cells. Nat Rev Immunol. 2010, 10: 170–181. 10.1038/nri2711 20154735

[45] Wu LH, Xu ZL, Dong D, He SA, Yu H. Protective effect of anthocyanins extract from blueberry on TNBS-induced IBD model of mice. Evid Based Complement Alternat Med. 2011, 2011: 525462 10.1093/ecam/neq040 21785630 PMC3135784

